# Mutation in DDM1 inhibits the homology directed repair of double strand breaks

**DOI:** 10.1371/journal.pone.0211878

**Published:** 2019-02-11

**Authors:** Seung Hee Choi, Tae Ho Ryu, Jeong-Il Kim, Sungbeom Lee, Seung Sik Lee, Jin-Hong Kim

**Affiliations:** 1 Advanced Radiation Technology Institute, Korea Atomic Energy Research Institute, Jeongeup-si, Jeollabuk-do, Republic of Korea; 2 Department of Biotechnology, Chonnam National University, Gwangju, Republic of Korea; 3 Department of Radiation Biotechnology and Applied Radioisotope Science, University of Science and Technology, Yuseong-gu, Daejeon, Republic of Korea; Tulane University Health Sciences Center, UNITED STATES

## Abstract

In all organisms, DNA damage must be repaired quickly and properly, as it can be lethal for cells. Because eukaryotic DNA is packaged into nucleosomes, the structural units of chromatin, chromatin modification is necessary during DNA damage repair and is achieved by histone modification and chromatin remodeling. Chromatin remodeling proteins therefore play important roles in the DNA damage response (DDR) by modifying the accessibility of DNA damage sites. Here, we show that mutation in a SWI2/SNF2 chromatin remodeling protein (DDM1) causes hypersensitivity in the DNA damage response via defects in single-strand annealing (SSA) repair of double-strand breaks (DSBs) as well as in the initial steps of homologous recombination (HR) repair. *ddm1* mutants such as *ddm1-1* and *ddm1-2* exhibited increased root cell death and higher DSB frequency compared to the wild type after gamma irradiation. Although the *DDM1* mutation did not affect the expression of most DDR genes, it did cause substantial decrease in the frequency of SSA as well as partial inhibition in the γ-H2AX and Rad51 induction, the initial steps of HR. Furthermore, global chromatin structure seemed to be affected by *DDM1* mutations. These results suggest that DDM1 is involved in the homology directed repair such as SSA and HR, probably by modifying chromatin structure.

## Introduction

DNA damage is caused by cellular metabolic processes such as oxidative respiration, or by toxic chemicals or environmental stresses such as UV/ionizing radiation [1–3]. Cells deal with DNA damage through a network of cellular pathways called the DNA damage response (DDR), as unrepaired damage can lead to genome instability and tumorigenesis [4, 5]. It is therefore very important for cells to have efficient, tightly controlled DNA damage response pathways.

One of the most dangerous types of DNA damage is double-strand breaks (DSBs), which are repaired by two main mechanisms; homology directed repair (HDR) and non-homologous end joining (NHEJ) [6]. HDR is mediated via single-strand annealing (SSA) and homologous recombination (HR). The latter HR repair includes double-strand break repair (DSBR), synthesis-dependent strand annealing (SDSA), and break-induced replication (BIR). While SSA is mediated by RAD52, HR repair requires RAD51 [7, 8]. RAD51-independent SSA is error-prone but suggested as the most efficient pathway of homology-dependent DSB repair [9]. HR is a very accurate DSB repair pathway that is available only in the late S and G2 phases of the cell cycle after cell DNA replication because of the need for sister chromatids as templates for break repair. In contrast, NHEJ occurs in all phases of the cell cycle, and directly joins two broken ends of DNA. During NHEJ, the joining of DNA ends with short homologous sequences can cause mutations such as nucleotide deletions, insertions, or translocations. NHEJ is therefore a more error-prone repair process than HR. Both HR and NHEJ mechanisms require DNA processing procedures controlled by post-translational modifications such as phosphorylation and ubiquitination of the chromatin and DNA damage repair proteins. At DSB sites, the histone H2A variant, H2AX, is phosphorylated by phosphatidylinositol 3-kinases ATM and ATR to recruit DNA damage repair proteins [5].

Given that eukaryotic DNA is organized into nucleosomes and chromosomes, nucleosome compaction prevents various catalytic enzymes from accessing their target DNA during DNA damage repair. Therefore, chromatin remodeling activities to increase the accessibility of DNA damage sites are critical for the removal of DNA lesions [10–12]. The chromatin remodeling mainly occurs via two mechanisms [13]; first, chromatin structure is altered by posttranslational modification of histones via the action of histone modification enzymes [11, 14, 15]. Second, chromatin structure is changed by the displacement of histones or entire nucleosomes via the action of ATP-dependent chromatin remodeling complexes and histone chaperones [16–18]. Among the ATP-dependent chromatin remodeling complexes, switch2/sucrose nonfermentable2 (SWI2/SNF2) chromatin remodeling factors have been implicated in the DSB repair pathways in a diverse range of organisms, including mammals [5, 14–16, 19, 20]. The SWI2/SNF2 chromatin remodeling factors can catalyze the sliding, eviction, or alteration in composition of nucleosomes and can change chromatin structure at DSB sites [16, 21].

In *Arabidopsis thaliana*, mutations in several SWI2/SNF2 proteins lead to hypersensitivity after exposure to DNA-damaging agents such as gamma radiation [22–24]. Among them, radiation-sensitive 54 (RAD54) and inositol auxotrophy 80 (INO80) play important roles in HR [25, 26] and PIE1, a subunit of SWR1 complex, functions in DNA damage repair, somatic recombination, and meiosis. Considering these roles, many other SWI2/SNF2 chromatin remodelers need to be further explored as putative regulators of DNA damage repair.

*Arabidopsis decrease in DNA methylation1* (*ddm1*) mutants were initially isolated as genomic DNA methylation-defective mutants [27, 28]. DDM1 is a member of the SWI2/SNF2 protein family, and is required for normal patterns of genomic DNA methylation in *Arabidopsis*. It allows DNA methyltransferases to access H1-containing heterochromatin, contributing to RNA-directed DNA methylation (RdDM) pathways for stable silencing of transposable elements [29]. The *ddm1* mutants have been reported to show increased sensitivity to gamma radiation, UV-C [22], UV-B [30], methyl methane sulfonate (MMS), and NaCl stresses [31]. Although DNA damage-sensitive phenotypes of *ddm1* mutants have been reported, the underlying mechanisms by which SWI2/SNF2 remodeling factor DDM1 participates in DSB repair remain to be elucidated.

Therefore, in this study, the potential involvement of DDM1 in DSB repair via SSA and HR was investigated in *ddm1* mutants by SSA frequency, γ-H2AX, and RAD51 assays after gamma irradiation to induce DSBs. Experimental results suggest that the SWI2/SNF2 chromatin remodeling protein, DDM1, is involved in the SSA and HR repair of DSBs.

## Materials and methods

### Plant materials and gamma irradiation

*Arabidopsis thaliana* (ecotype Columbia WT, *ddm1-1*, and *ddm1-2* mutants) were grown in a controlled growth room at 100–130 μmol m^–2^ s^–1^ and a 16-h photoperiod at 22/18°C (day/night). *Arabidopsis* seedlings were grown on 1× MS (pH 5.7) agar plates with 3% sucrose. The *ddm1-1* mutant and the DGU.US reporter were kindly provided by Dr. G. Eric Schaller and Dr. Holger Puchta, respectively. The *ddm1* mutants and GUS-based DGU.US HR reporter line have also been described previously [9, 27, 28]. The reporter line was crossed with the *ddm1* mutants, and double homozygote lines were obtained in F3 and F4 populations through PCR-based genotyping using the restriction endonucleases *Nsi*I and *Rsa*I, which identify single-nucleotide substitutions. *DDM1*-overexpressing transgenic plants were generated in WT and *ddm1* mutant backgrounds using the pK7WG2.0 vector by the floral-dip method [32].

Five-day-old seedlings were irradiated with 80, 200, and 500 Gy of gamma radiation at dose rates of 20, 50, 125 Gy h^–1^ for 4 h, or 480, 1,200, 3,000 Gy h^–1^ for 10 min using a ^60^Co gamma irradiator (IR-222, MDS Nordion Inc., Kanata, Canada). For root cell death assays, root swelling assays, and comet assays, the plants were irradiated with 200 Gy of gamma radiation at a dose rate of 50 Gy hr^–1^ for 4 h. To check the phenotypes, the irradiated plants were further incubated in the growth room for the indicated number of days after gamma irradiation.

### RNA isolation and microarray analysis

Total RNA was isolated and mixed from three biological replicates of two-week-old WT and *ddm1-2* mutant seedlings after gamma irradiation using the RNeasy Mini Kit (Qiagen, Hilden, Germany) according to the manufacturer’s instructions. The RNA was reverse-transcribed and amplified, and then hybridized onto the Agilent Arabidopsis (V4) Gene Expression Microarray, 4x44K (Agilent Technologies, Palo Alto, CA). All experimental procedures, including RNA preparation, quality check, conversion into double-stranded cDNA, generation of biotin-labeled cRNA, and hybridization onto the genome array, were performed according to protocols provided by Agilent. We compared the transcriptome data between 200 Gy-treated WT and 200 Gy-treated *ddm1-2*, and selected genes that were induced or repressed at significant levels (more than 2-fold). The top 10 gene ontology (GO) terms (Table 1) were selected based on *P* values using DAVID [33] software to identify cellular processes affected by *DDM1* mutation after gamma irradiation.

**Table 1:** Top 10 gene ontology (GO) terms in the biological process category affected by gamma irradiation.

GO-ID	GO-term	k^a^	*P* Value	C^b^
***Up-regulation***				
GO:0016042	Lipid catabolic process	21/1151 (1.8%)	0.0001	275/27416 (1%)
GO:0019953	Sexual reproduction	6/1151 (0.5%)	0.0017	227/ 27416 (0.8%)
GO:0071281	Cellular response to iron ion	7/1151 (0.6%)	0.0018	35/27416 (0.1%)
GO:0031146	SCF-dependent proteasomal ubiquitin-dependent protein catabolic process	13/1151 (1.1%)	0.0036	16/27416 (0.1%)
GO:0050832	Defense response to fungus	25/1151 (2.2%)	0.0060	561/27416 (2%)
GO:0055114	Oxidation-reduction process	61/1151 (5.3%)	0.0078	5166/27416 (18.8%)
GO:0006417	Regulation of translation	7/1151 (0.6%)	0.0327	252/27416 (0.9%)
GO:0019745	Pentacyclic triterpenoid biosynthetic process	3/1151 (0.3%)	0.0407	13/27416 (0.05%)
GO:0071281	Cellulose catabolic process	4/1151 (0.3%)	0.0524	27/27416 (0.1%)
GO:0043086	Negative regulation of catalytic activity	6/1151 (0.5%)	0.0558	401/27416 (1.5%)
***Down-regulation***				
GO:0000165	MAPK cascade	12/1089 (1.1%)	0.0001	129/27416 (0.5%)
GO:0009607	Response to biotic stimulus	10/1089 (0.9%)	0.0001	1983/27416 (7.2%)
GO:0016042	Lipid catabolic process	16/1089 (1.5%)	0.0019	275/27416 (1%)
GO:0007166	Cell surface receptor signaling pathway	8/1089 (0.7%)	0.0028	375/27416 (1.4%)
GO:0006468	Protein phosphorylation	51/1089 (4.7%)	0.0043	3184/27416 (11.6%)
GO:0006855	Drug transmembrane transport	9/1089 (0.8%)	0.0059	100/27416 (0.4%)
GO:0009308	Amine metabolic process	4/1089 (0.4%)	0.0078	274/27416 (1.0%)
GO:0006351	Transcription, DNA-templated	95/1089 (8.7%)	0.0084	5502/27416 (20.1%)
GO:0050832	Defense response to fungus	30/1089 (2.8%)	0.0093	561/27416 (2%)
GO:0032259	Methylation	17/1089 (1.6%)	0.0094	907/27416 (3.3%)

Gene ontology analysis was performed using radiation-responsive transcripts showing a more than 2-fold difference in expression between the WT and *ddm1-2* mutant after gamma irradiation. GO terms in the table were selected based on P values in each GO category.

^a^Cluster frequency of a given GO term in the differentially expressed genes selected. Numbers in parentheses indicate percentages.

^b^The total frequency of a given GO term in all annotations in the Arabidopsis genome.

For qRT-PCR, first-strand cDNA was produced using the ImProm II Reverse Transcriptase system kit (Promega, Madison, WI). PCR reactions were then performed in a CFX 96 Real-Time PCR System (Bio-Rad, Foster City, CA) using the QuantiTect SYBR Green PCR Kit (Qiagen, Hilden, Germany). The gene-specific primers for PCR reactions are described in S1 Table. The template cDNA was initially activated at 95°C for 15 min, amplified by 40 cycles at 94°C for 15 s and 58°C for 30 s, then extended at 72°C for 30 s. The relative expression level of each gene was calculated between the control and gamma-irradiated samples using the comparative C_T_ method [34]. *ACTIN2* was used as an endogenous control gene to normalize for differences in the amount of total DNA.

### Root cell death and root swelling assays

Cell death assays were performed on root tips as previously described [35] with slight modifications. Root tips were immersed in propidium iodide solution (5 μg ml^–1^) for 1 min and rinsed three times with deionized water. We observed stained samples with a confocal laser scanning microscope (LMS-800, Carl Zeiss, Jena, Germany), and measured the area of dead cells in the root meristem with ImageJ 1.49v software (NIH, Bethesda, MD, USA). The proportion of cell death was obtained by dividing the dead cell area by the root meristem area. For the root swelling assay, primordial root tips were observed using a bright-field microscope (BX50, Olympus, Tokyo, Japan) at 8 days after gamma irradiation.

### Comet assay

Approximately 100 mg of seedlings were dissected with a razor blade in 1× phosphate-buffered saline supplemented with 50 mM EDTA on ice. The released nuclei were separated from the cell debris by centrifugation, and the suspension was thoroughly mixed with an equal volume of warm 1% low-melting-point agarose at 40°C. The comet assay was performed under neutral (N/N) conditions following the standard protocol [36]. Air-dried slide samples were stained with propidium iodide solution (2.5 μg ml^–1^). More than 150 cells per sample were analyzed from three biological replicates, and the percentage of DNA in tails was calculated using the image analysis system (Komet 5.5 from Kinetic Imaging Ltd., Liverpool, UK).

### γ-H2AX and RAD51 assays

Seedlings were harvested 30 min after gamma irradiation and frozen immediately with liquid nitrogen. The seedlings were ground with a mortar and pestle and resuspended in nuclear isolation buffer (0.25 M sucrose, 60 mM KCl, 15 mM NaCl, 5 mM MgCl_2_, 1 mM CaCl_2_, 15 mM PIPES pH 6.8, 0.8% Triton X-100, 1 mM PMSF) with protease inhibitor cocktail (Roche Diagnostics, Mannheim, Germany) and phosphatase inhibitor (50 mM Na_3_VO_4_ and 30 mM NaF). The suspension was filtered twice through Miracloth, and the filtrate was centrifuged at 10,000 × *g* for 20 min at 4°C. The pellet was resuspended in 1ml of 0.4 M H_2_SO_4_ and left on ice for 1 h. This suspension was centrifuged at 15,000 × *g* for 5 min at 4°C and then the soluble proteins were precipitated from the supernatant with 12 volumes of acetone at –20°C. The precipitate was collected by centrifugation at 7,000 × *g* for 15 min at 4°C, and the pellet was then resuspended in 4 M urea. The protein samples were separated by 15% sodium dodecyl sulfate-polyacrylamide gel electrophoresis and electro-transferred to polyvinylidene fluoride membranes. For γ-H2AX assays, rabbit anti-human γ-H2AX H5912 antibodies (1:1,000; Sigma-Aldrich, St. Louis, MO, USA) were used to detect γ-H2AX as previously described [37].

RAD51 assays were performed as previously described [38] with slight modifications. Seedlings were harvested 30 min after gamma irradiation and frozen immediately with liquid nitrogen. The protein samples were separated by 15% sodium dodecyl sulfate-polyacrylamide gel electrophoresis and electro-transferred to polyvinylidene fluoride membranes. Immunostaining for RAD51 was performed using a rabbit anti-Rad51-ab48981 antibody (Abcam, Cambridge, MA, USA) at a dilution of 1:1000. HRP goat anti-rabbit antibody (Life Technologies, Carlsbad, CA, USA) was used as a secondary antibody at a 1:3000 dilution.

### SSA assay

Fourteen-day-old DGU.US seedlings were irradiated with 200 Gy of gamma radiation at a dose rate of 1,200 Gy h^–1^ for 10 min, then incubated in a growth room for 4 days. The seedlings were incubated overnight in a GUS staining solution including 100 mM NaHPO_4_ (pH 7.0), 0.5 mM K_4_Fe(CN)_6_, 0.5 mM K_3_Fe(CN)_6_, 10 mM EDTA, 0.08% 5-bromo-4-chloro-3-indolyl-β-D-glucuronide (X-Gluc), and 0.05% Triton X-100. After destaining with ethanol, the average number of blue spots per sample (40 plants) was determined from three biological replicates (a total of 120 samples) using a Leica EZ4E microscope (Leica, Heerbrugg, Switzerland). This number of blue spots was taken to represent the frequency of SSA in the WT and *ddm1* mutant lines.

### MNase assay

MNase assays were performed as previously described [39]. MNase-digested chromatin DNA was electrophoresed on 1.5% agarose gels and visualized by staining with Gelred (41003, Biotium, Fremont, CA). The genomic band intensities without (g_0_) and with (g_c_) treatment with different concentrations of MNase were quantified using Image J software (NIH, Bethesda, MD, USA). The ratio of g_c_/g_0_ was used to represent the degree of chromatin relaxation.

## Results

### Mutations in a SWI2/SNF2 gene, *DDM1*, lead to hypersensitive phenotypes in *Arabidopsis* after gamma irradiation

**Fig 1:**
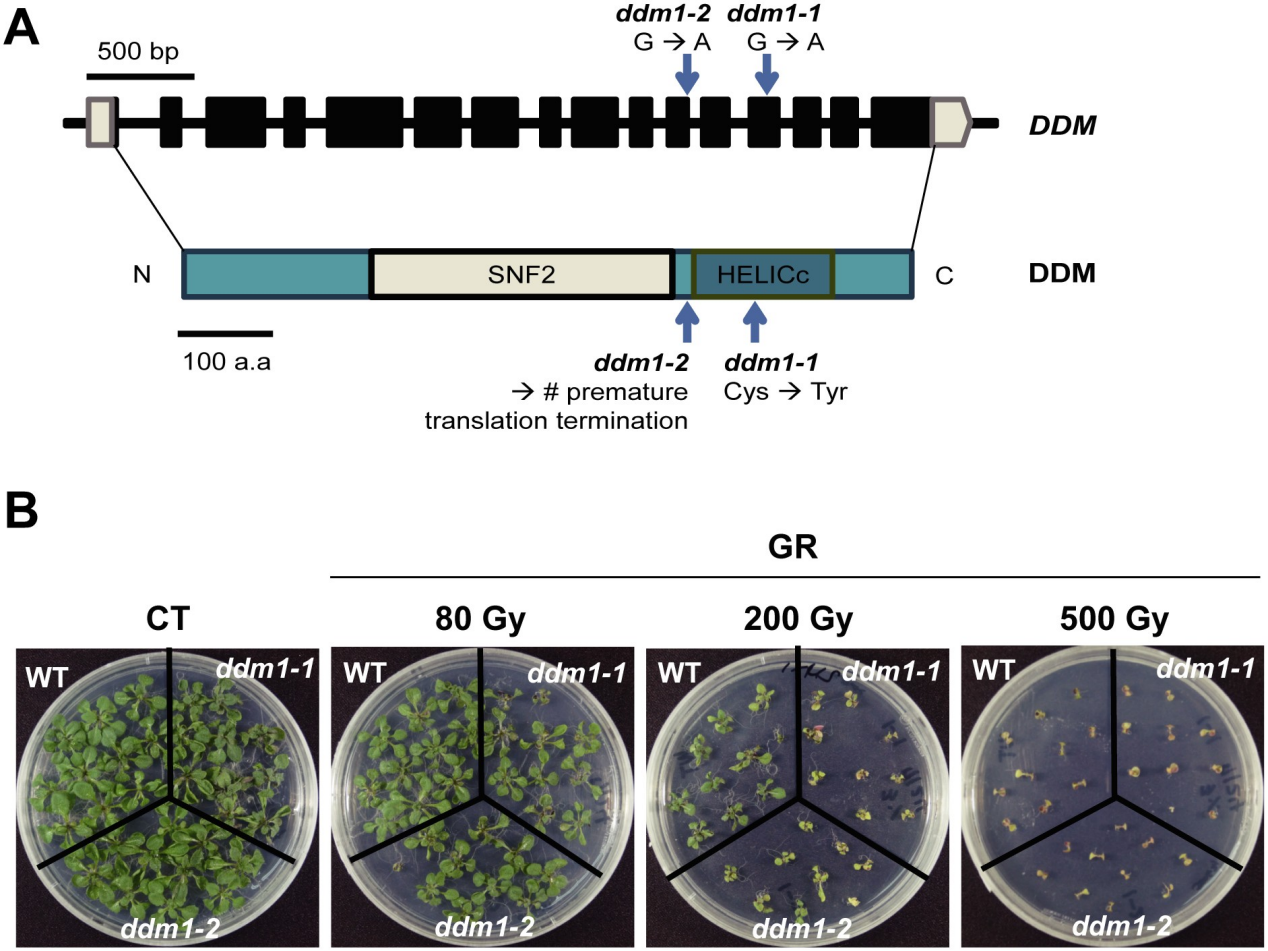
*ddm1* mutants show hypersensitive phenotypes after gamma irradiation. (A) Physical map of *DDM1* and its mutations in *ddm1* mutants. (B) Phenotypes of Col-0 (WT) and *ddm1* mutants after exposure to gamma radiation. Images were taken 14 days after gamma irradiation. CT, control; GR, gamma radiation. Digits in parenthesis indicate a dose of gamma radiation.

*DDM1* is a homolog of the yeast *RAD54* gene, and plays diverse roles in *Arabidopsis*. RAD54 is known to be a member of the SWI2/SNF2 family, which is involved in DNA damage repair and development in many organisms [40–42]. Forty-one SWI2/SNF2 proteins have been identified in *Arabidopsis* based on yRAD54 homologous sequence searches [22–24]. Previous studies revealed hypersensitive phenotypes of some *Arabidopsis* SWI2/SNF2 mutants to DNA-damaging agents, as shown by the reduced number of true leaves in *ddm1-2* and *ddm1-5* mutants after gamma irradiation [22]. Similarly, we observed radiation-sensitive phenotypes in two *ddm1* mutants, *ddm1-1* and *ddm1-2*, depending on radiation dose (Fig 1). Although mammals have a lethal dose below tens of Gy, plants can survive hundreds of Gy to induce DNA damage response [43]. After exposure to gamma radiation at 200 Gy, the *ddm1-1* and *ddm1-2* mutants exhibited remarkably small and abnormal phenotypes compared to the wild type (WT). However, radiation doses of 80 Gy or 500 Gy seemed to be too low or too high, respectively, to differentiate the radiation sensitivity between the WT and *ddm1* mutants. These results suggest that *DDM1* mutation can induce various hypersensitive phenotypes in *Arabidopsis* upon exposure to gamma radiation.

**Fig 2:**
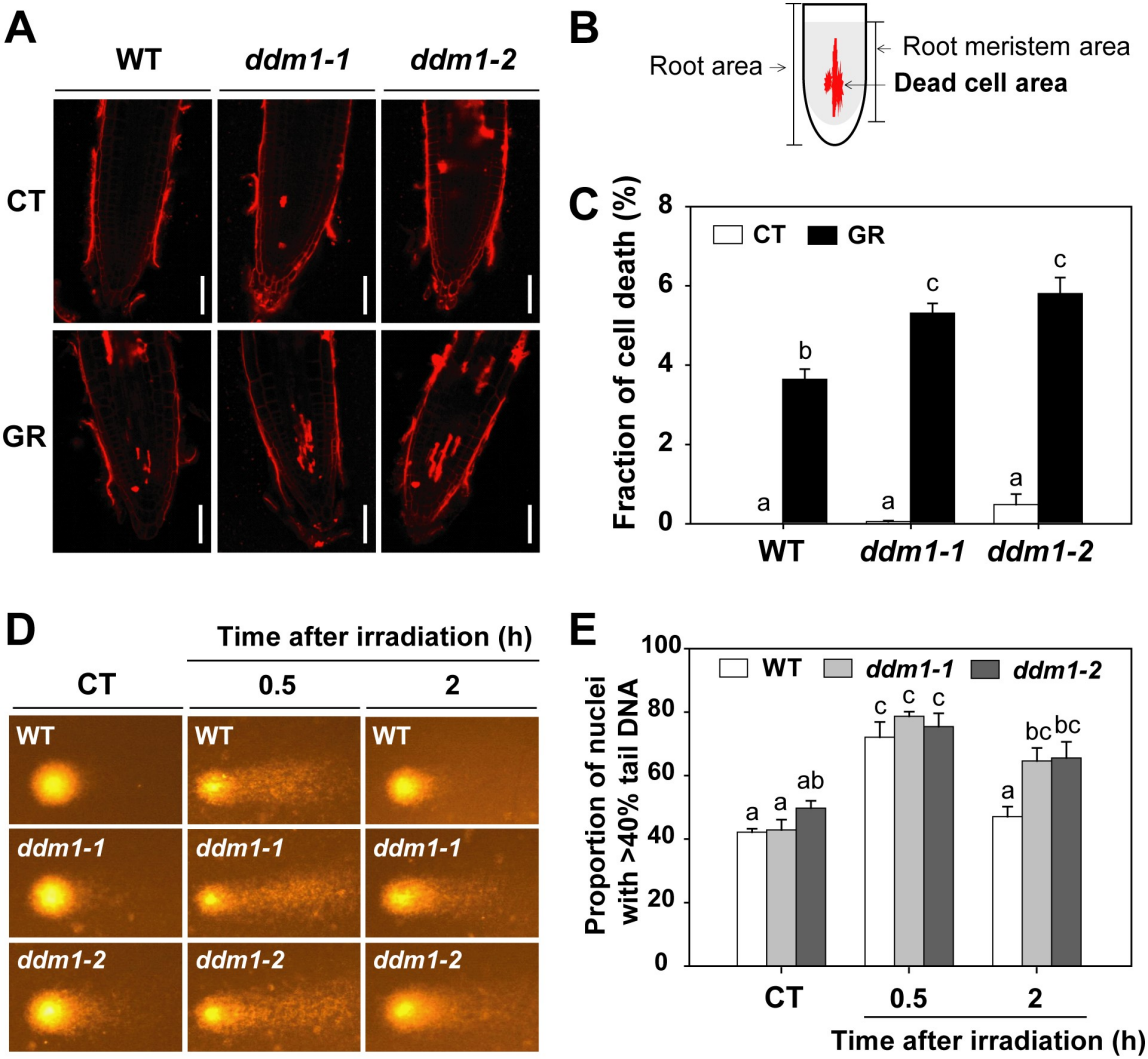
*DDM1* mutation increases cellular DNA damage upon exposure to gamma radiation. (A) Representative images of root tips, which were stained with propidium iodide 1 day after gamma irradiation. Scale bars: 50 μm. (B) Diagram to show extent of dead cell area. (C) Proportion of dead cell area in root meristem. Bars represent means ± SE (n = 19) from three independent experiments. (D) Representative comet images of nuclei and (E) proportion of nuclei with more than 40% tail DNA in WT and *ddm1* mutants without or with exposure to 200 Gy of gamma radiation. Bars represent means ± SE (n = 3) of three independent experiments using 250 nuclei. Different letters in (C) and (E) indicate significant differences among the samples at a threshold of P < 0.05 [one-way ANOVA, Tukey’s honestly significant difference (HSD) test]. CT, control; GR, gamma radiation.

We further analyzed the radiation-sensitive phenotypes of *ddm1* mutants to correlate cellular DNA damage levels with root cell death, root swelling, and comet (single cell gel electrophoresis) assays. Upon exposure to gamma radiation, cell death of root meristematic tissues was significantly higher in the *ddm1-1* and *ddm1-2* mutants than in the WT (Fig 2A and 2C). However, root morphology after gamma irradiation did not differ substantially between the WT and the *ddm1* mutants (S1 Fig). Neutral comet assays can detect DSBs [44], and the proportion of DNA in the tail reflects the degree of DNA damage in the nucleus [45]. The neutral comet assay revealed no substantial difference in nuclear DNA damage between the WT and *ddm1* mutants under control condition and 30 min after gamma irradiation (Fig 2D and 2E). However, the *ddm1* mutants displayed significantly higher proportions of nuclei with the increased tail DNA 2 h after gamma irradiation compared to the WT (Fig 2E). These results suggest that *DDM1* mutation causes hypersensitivity to gamma radiation in *Arabidopsis*, probably via defects in DSB repair.

### Transcriptional changes of DDR genes induced by gamma irradiation do not differ significantly between the WT and *ddm1* mutants

**Fig 3:**
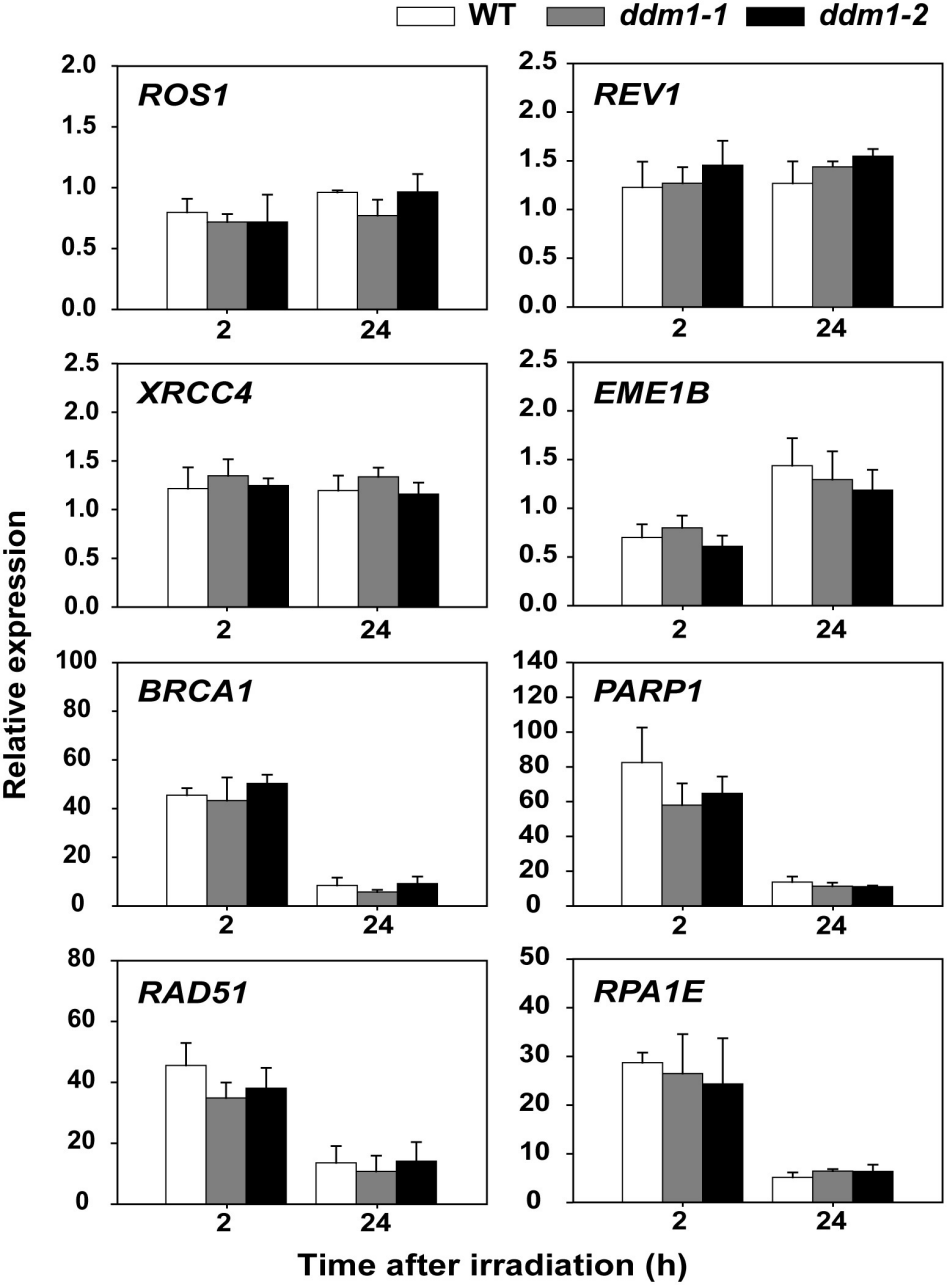
Radiation-sensitive phenotypes of *ddm1* mutants are not due to the altered transcription of DDR genes. Quantitative real-time PCR analysis of the expression of DDR genes in WT and *ddm1* mutants 2 and 24 h after gamma irradiation. *BRCA1*, breast cancer 1; *RPA1E*, replication protein A 1e; *PARP1*, poly [ADP-ribose] polymerase 1; *RAD51*, radiation sensitive 51. Bars represent means ± SE (n = 3) of three independent experiments. *ACTIN2* was used as an endogenous control gene.

Many SWI2/SNF2 chromatin remodeling complexes participate in transcriptional regulation [46, 47]. To investigate how DDM1 participates in DSB repair, we first analyzed the transcriptomes of the WT and the *ddm1-2* mutant after gamma irradiation using the Agilent *Arabidopsis* (V4) Gene Expression Microarray. We identified 3,507 probes significantly induced or repressed (more than 2-fold) by *DDM1* mutation. Through gene ontology (GO) analysis of the 3,507 probes using DAVID software [33], the top ten GO terms in the biological process category were selected based on the *P* values (Table 1). These GO terms were not directly related to the DDR, indicating that transcriptional changes in DDR genes after gamma radiation do not differ substantially between the WT and the *ddm1* mutants. Only four DNA damage repair genes (*REV1*, *EME1B*, *DML1*, and *XRCC4*) were identified among the 3,507 probes (S1 File). However, other representative DNA damage repair genes (e.g., *BRCA1*, *RAD51*, *RPA1E*, and *PARP1*) involved in HR [48], base excision repair (BER), and/or SSB repair [49] exhibited no significant transcriptional differences between the WT and the *ddm1* mutants (Fig 3). These results demonstrate that the pronounced DNA damage associating with the radiation-sensitive phenotypes in the *ddm1* mutants cannot be attributed to the altered transcription of DDR genes, including DNA damage repair genes.

### The *DDM1* mutation inhibits SSA repair of DSBs after gamma irradiation

**Fig 4:**
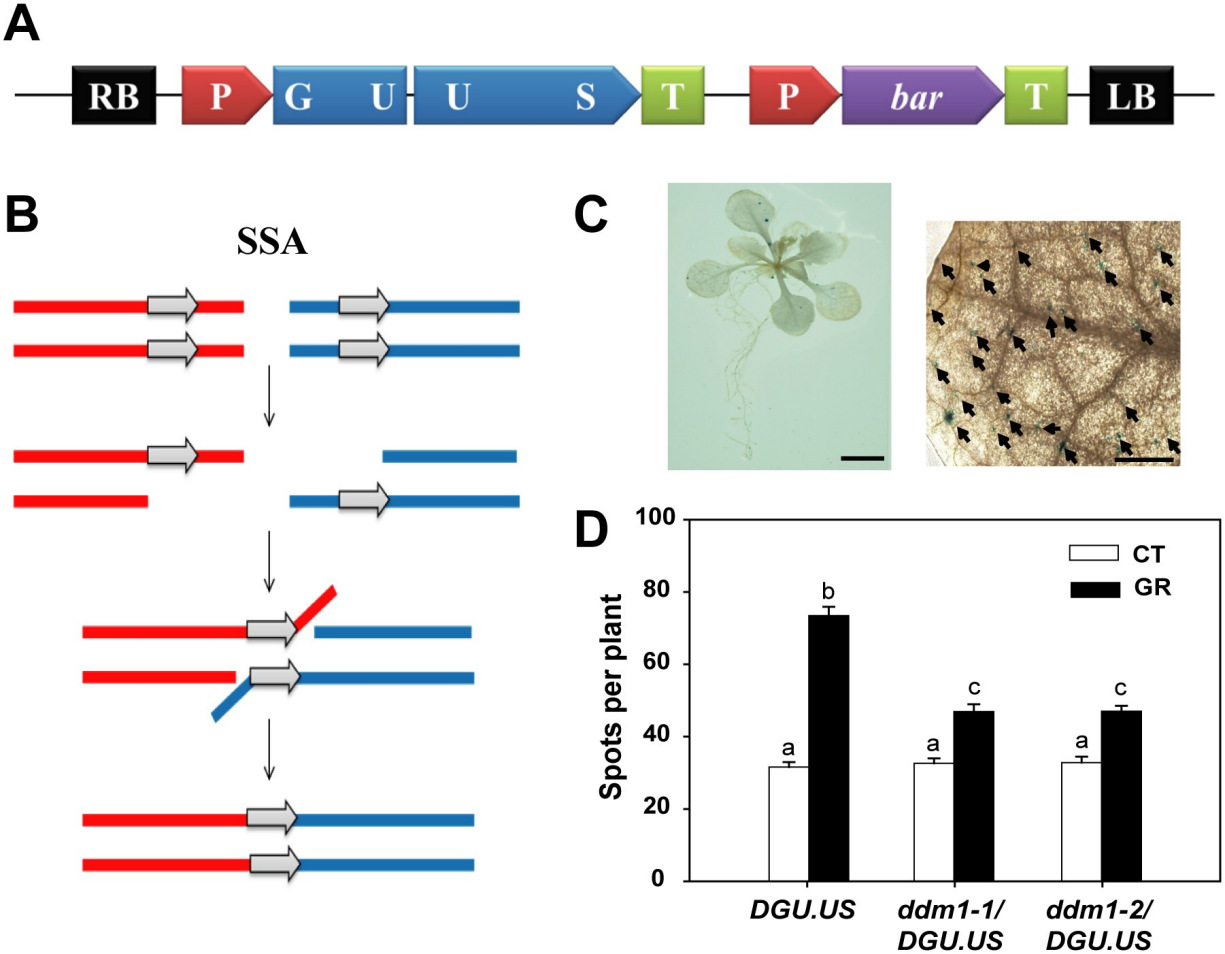
*DDM1* mutation causes defects in SSA repair of DSBs after gamma irradiation. (A) Physical map of the DGU.US-1 construct. (B) Model of SSA pathway for repair of DSBs (modified from [8, 9]). (C) Images of a whole seedling and a leaf showing blue spots, which represent SSA events in the reporter DGU.US line. Scale bars: 5 and 0.5 mm, respectively. (D) Change in the frequency of SSA events in DGU.US and *ddm1*/DGU.US lines after gamma irradiation. Bars represent means ± SE (n = 120) of three independent experiments. Different letters indicate significant differences among the samples at a threshold of P < 0.05 (one-way ANOVA, Tukey’s HSD test). CT, control; GR, gamma radiation.

DSBs are repaired by two major DNA damage repair mechanisms, HDR and NHEJ [6]. HDR includes SSA as well as HR repair consisting of DSBR, SDSA, and BIR. While SSA is mediated by RAD52, HR is dependent on RAD51 [7, 8]. RAD51-independent SSA seems to be error-prone but the most efficient pathway of homology-dependent DSB repair [9]. To test whether the chromatin remodeler DDM1 is involved in SSA repair of DSBs, therefore, we measured the frequency of SSA events using a transgene reporter DGU.US line, which contains two disrupted regions (GU and US) of the beta-glucuronidase (GUS) gene with a donor sequence in direct orientation (Fig 4A). The functional GUS gene is restored by SSA events, which take place in the overlapping region of the two disrupted parts (Fig 4B) [9]. Thus, SSA events can be visualized by blue spots corresponding to GUS activity in the transgenic plants (Fig 4C). When *ddm1* mutant homozygotes harboring the DGU.US reporter were compared with the reporter line under control conditions, the frequency of SSA events was comparable for both groups, being about 32 per plant on average (Fig 4D). However, after gamma irradiation this frequency increased up to 2.3-fold in the reporter line, but up to only 1.3-fold in the *ddm1*/DGU.US mutants, indicating a significant difference in SSA repair of DSBs between the WT and *ddm1* mutants. These results suggest that the DDM1 plays an important role in SSA events for DSB repair in *Arabidopsis* after gamma radiation.

### The *DDM1* mutation inhibits γ-H2AX and RAD51 induction, the initial steps of HR repair

**Fig 5:**
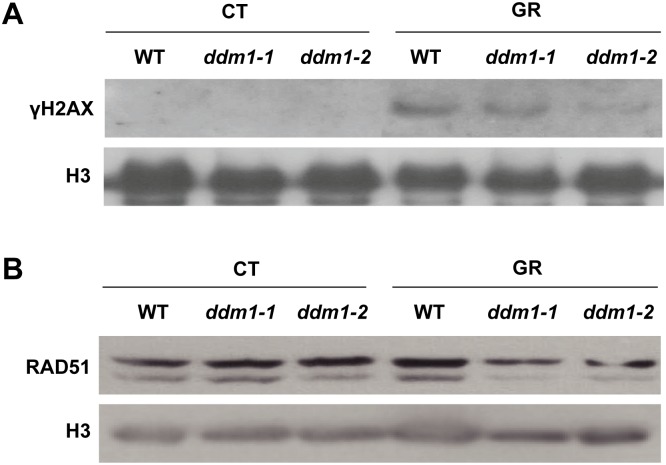
*ddm1* mutants have defects in induction of γ-H2AX and RAD51. (A) Immunoblot of γ-H2AX and (B) RAD51 in WT and *ddm1* mutants after gamma irradiation. CT, control; GR, gamma radiation.

Next, we attempted to investigate whether or how DDM1 affects HR repair in response to gamma radiation. It has been reported that chromatin remodelers, such as RAD54 and INO80, participate in HR in a range of organisms, including *Arabidopsis* [25, 26, 40]. Some SWI2/SNF chromatin remodelers are involved in γ-H2AX induction for efficient DSB repair in diverse organisms [50–53]. Considering the absence of a causal relationship between *DDM1* mutation and DDR gene expression (Table 1 and Fig 3), DDM1 may affect HR repair via chromatin remodeling rather than by transcriptional regulation of DDR genes for DSB repair. This speculation prompted us to test whether DDM1 plays a role in γ-H2AX induction to initiate HR repair. Immunoblot analysis of γ-H2AX showed that the H2AX phosphorylation increased markedly in the WT after gamma irradiation, but was substantially inhibited (by about 40–60%) in the *ddm1* mutants (Fig 5A). Therefore, H2AX phosphorylation seems to be significantly impaired by *DDM1* mutation, suggesting a potential role for DDM1 in the efficient induction of H2AX phosphorylation upon exposure to gamma radiation. RAD51 is also regarded as a prerequisite for the initiation of HR [8, 54–57]. RAD51 is a homolog of the bacterial recombinase RecA; it facilitates a physical connection between the invading DNA substrate and homologous duplex DNA template in eukaryotic cells [58]. To reveal whether DDM1 plays a role in RAD51 induction to initiate HR repair, we compared the level of RAD51 proteins between the WT and *ddm1* mutants upon exposure to gamma radiation. It has been reported that loss of RAD51 proteins bring about hypersensitivity to DNA-damaging agents, such as bleomycin and cisplatin [39, 59–62]. In agreement with their crucial role in HR, we found that RAD51 also decreased in the *ddm1-1* and *ddm1-2* mutants after gamma irradiation (Fig 5B). These results suggest that the HR repair in the *ddm1* mutants could be somewhat affected by defects in RAD51 induction, as well as γ-H2AX induction.

### *DDM1* mutation has influence on the structural changes in chromatin after gamma irradiation

**Fig 6:**
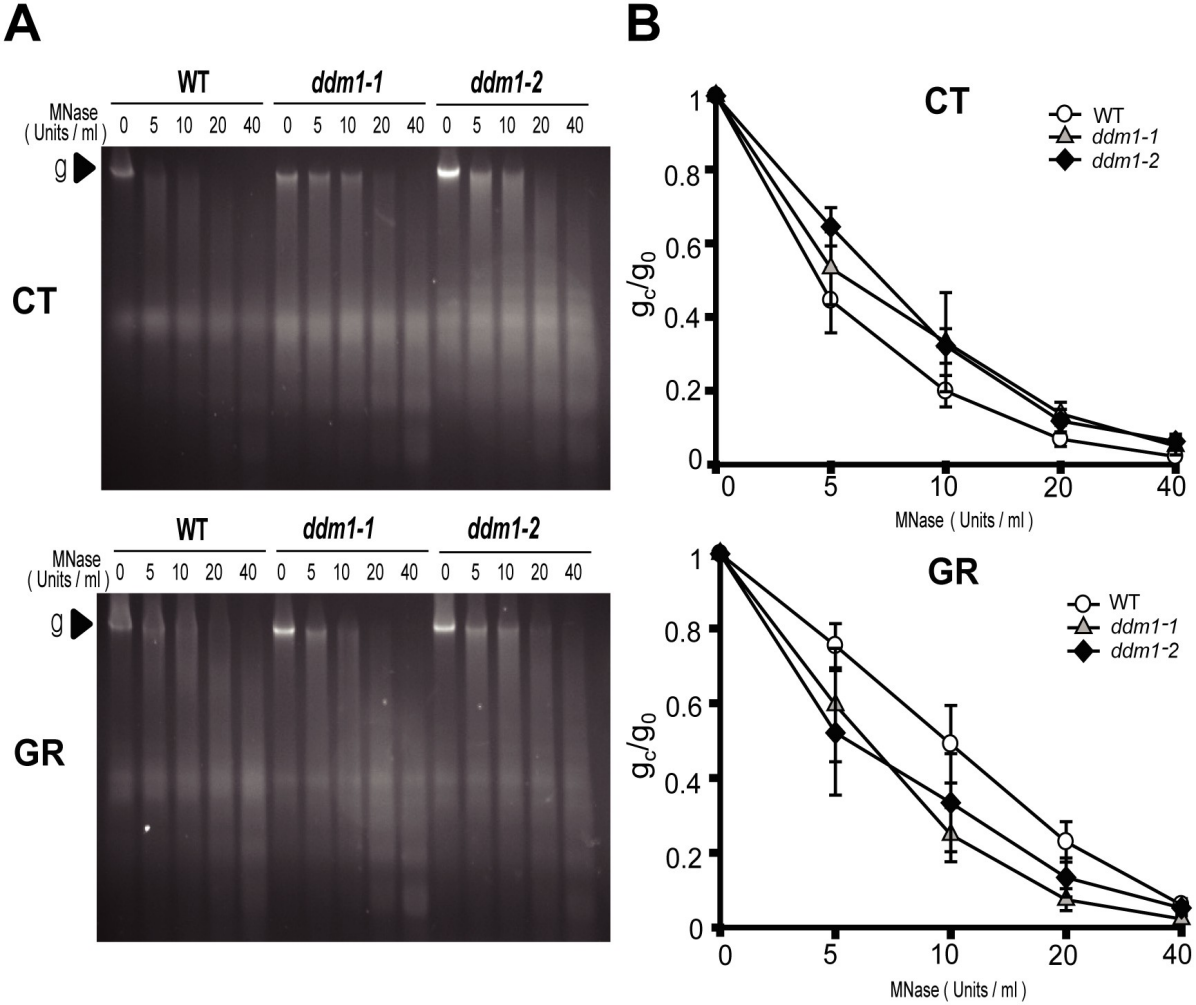
*DDM1* mutation affects chromatin structure before and after gamma irradiation. (A) Chromatin digestion with micrococcal nuclease (MNase). (B) Proportion of MNase-digested chromatin DNA resolved on agarose gels. The genomic band intensities without (g_0_) and with (g_c_) treatment with different concentrations of MNase were quantified, and the ratio of g_c_/g_0_ was used to represent the degree of chromatin relaxation. Bars represent means ± SE (n = 3) of three independent experiments. CT, control; GR, gamma radiation.

Since the packaging of eukaryotic DNA into chromatin restricts the recruitment of DNA damage-repair machinery to DNA damage sites, potential relationships between chromatin remodeling and DNA damage repair have been explored in many organisms, from yeast to human [13, 16, 24]. Accordingly, we investigated the effects of *DDM1* mutation on chromatin structure by a chromatin accessibility test using micrococcal nuclease (MNase) that cuts inter-nucleosomal DNA [63]. The ratios of intact genomic band intensities treated with different concentrations of MNase (g_c_) to the non-treated control (g_0_) represent the degree of chromatin relaxation. Chromatin accessibility in *ddm1* mutants under control conditions was slightly lower than that in the WT, as revealed by the higher g_0_/g_c_ ratio. However, chromatin accessibility in *ddm1* mutants was higher after gamma irradiation (Fig 6). These results indicate that *DDM1* mutation has influence on the chromatin structure, and may influence DNA damage repair by affecting chromatin remodeling after exposure to gamma radiation.

### Complementation of *DDM1* mutation rescues DNA damage-sensitive phenotypes in *ddm1* mutants

**Fig 7:**
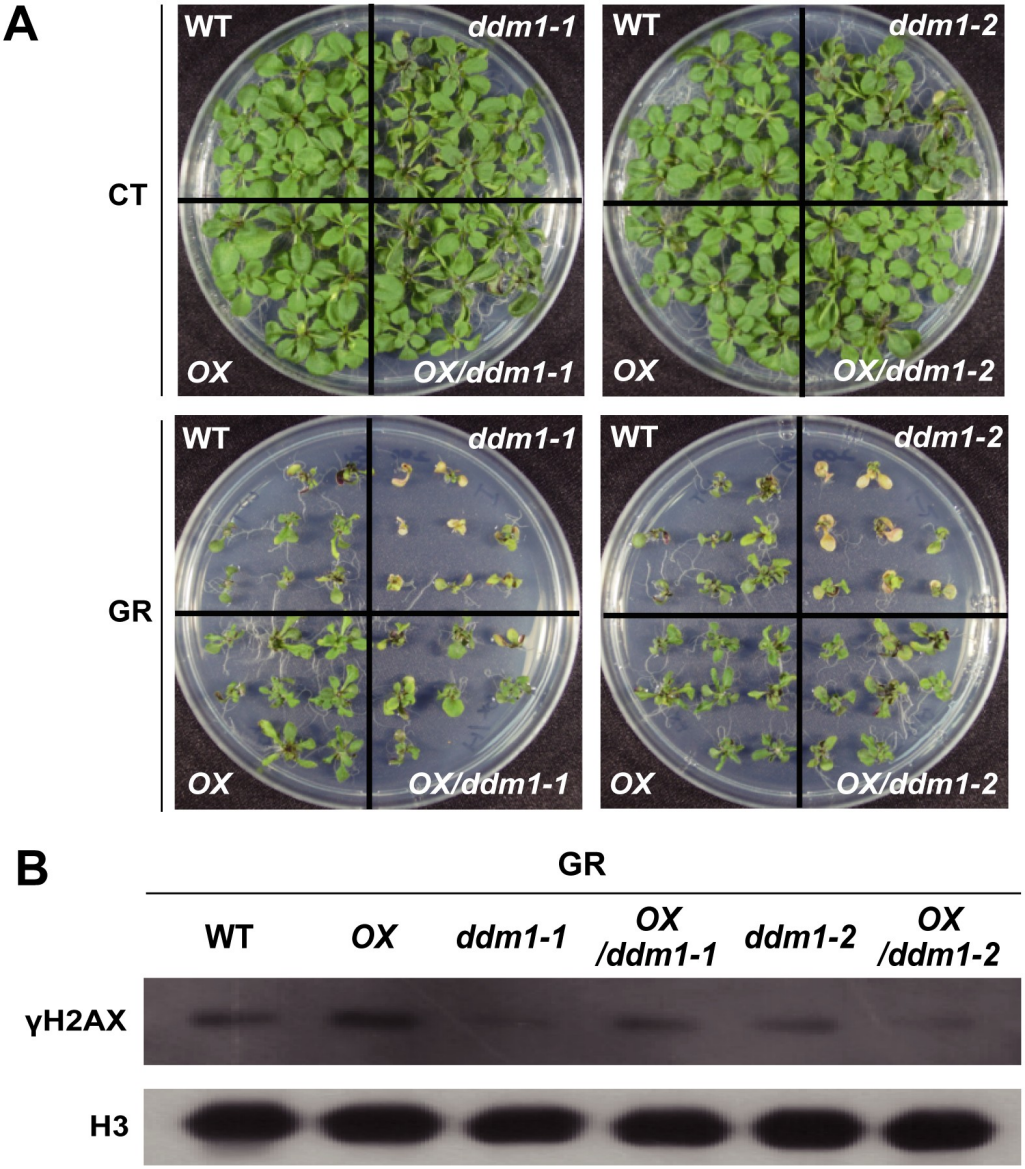
Complementation of *DDM1* mutation by overexpressing the full-length cDNA of *DDM1* in the ddm1 mutant is effective. (A) Phenotypes of transgenic lines overexpressing the *DDM1* cDNA in WT and *ddm1* mutant backgrounds in response to gamma irradiation. Seedlings were grown for 18 days after gamma irradiation at 200 Gy for 4 h. CT, control; GR, gamma radiation. (B) Immunoblot of γ-H2AX in transgenic lines overexpressing the *DDM1* cDNA in WT and *ddm1* mutant backgrounds after gamma irradiation at 200 Gy.

To reveal whether intact DDM1 is required for normal DNA damage response in *Arabidopsis*, we generated transgenic lines overexpressing the *DDM1* cDNA in WT and *ddm1* mutant backgrounds. In control, overexpression (OX) lines in *ddm1* mutants did not exhibit any distinct or abnormal phenotypes compared to *ddm1* mutants. However, when we checked the DNA damage sensitive phenotypes after gamma irradiation, DNA damage-sensitive phenotypes in *ddm1* mutants were recovered in OX / *ddm1* mutants like WT phenotypes (Fig 7A). We further analyzed the H2AX phosphorylation after gamma irradiation. Immunoblot analysis of γ-H2AX showed the significantly increased H2AX phosphorylation in DDM1 OX than in the WT (Fig 7B). In addition, the substantially decreased H2AX phosphorylation in the *ddm1-1* mutants was recovered in OX / *ddm1-1* albeit lesser γ-H2AX in OX / *ddm1-2*. These results imply that the DNA damage-sensitive phenotypes and decreased γ-H2AX in the *ddm1* mutants are caused by loss of functional DDM1 and DDM1 is required for normal DNA damage response in *Arabidopsis*.

## Discussion

It has been reported that *Arabidopsis* SWI2/SNF2 chromatin remodeling genes are involved in the DDR, including HR repair [22]. *ddm1* mutants exhibit hypersensitive phenotypes in response to DNA-damaging agents such as gamma radiation, UV, and MMS [30, 31]. Although *DDM1* knockdown did not affect intrachromosomal recombination frequency in *Arabidopsis* [22], DDM1 might be involved in other types of DSB repair not previously tested. HDR mechanisms for DSB repair are known to include SSA as well as HR such as DSBR, SDSA, and BIR. Therefore, to substantiate the possibility that DDM1 plays a role in HDR like yRAD51, we used the DGU.US reporter line that can be restored by SSA [9]. SSA repair associated with deletions is error-prone but the most efficient pathway of homology-dependent DSB repair. Our results demonstrated that *DDM1* mutation inhibits SSA events after gamma irradiation (Fig 4). Next, we investigated whether DDM1 is involved in the phosphorylation of H2AX and proper generation of RAD51, the initial steps for HR repair. *DDM1* mutation decreased both the γ-H2AX and RAD51 levels after gamma irradiation (Fig 5). H2AX proteins are phosphorylated within minutes of DNA damage, then spread to flank the DSB sites and serve as a signal for the recruitment of DSB repair machinery [64, 65]. The mammalian SWI2/SNF2 complexes were suggested to facilitate H2AX phosphorylation by influencing the higher-order chromatin structure in such a way as to increase the accessibility of the H2AX-containing nucleosomes [19]. Therefore, the reduced level of γ-H2AX in the *ddm1* mutants may be attributed to the misconducted structural change of chromatin to initiate DSB repair. Second, RAD51 is homologous to bacterial RecA recombinase and a key factor in HR [55, 59]. RAD51 proteins search for homologous DNA strands and join single-stranded DNA to the homologous DNA template strand via ATP hydrolysis, leading to DSB repair [58, 66]. Loss of RAD51 sensitizes plants toward DNA-damaging agents, such as bleomycin and cisplatin [39, 59–62]. Considering the importance of γ-H2AX and RAD51 induction in DSB repair with the fact that RAD51 proteins are recruited to γ-H2AX sites after DNA damage [67, 68], it is suggested that *DDM1* mutation may affect HR repair of DSBs by inhibiting the generation of RAD51 foci as well as the phosphorylation of H2AX. This possibility needs to be substantiated in a further study to evaluate SDSA events of HR repair in the *ddm1* mutants.

DDM1 functions as a chromatin remodeling factor *in vitro* [69] and is required for remodeling heterochromatic, H1-bound nucleosomes to facilitate access of DNA methyltransferases in the RdDM pathway [29]. The DNA damage-sensitive phenotypes in the *ddm1* mutants could be initially attributed to defects in DNA methylation. However, mutations in *MET1*, a cytosine methyltransferase gene, did not induce DNA damage-sensitive phenotypes [22]. Similarly, other RdDM mutants such as *cmt3*, *rdr6*, *drm2*, and *dcl3*, which have defects in maintenance of DNA methylation levels, also exhibited no sensitive phenotypes after gamma irradiation (S2 Fig). Although DNA hypomethylation or hypermethylation has been associated with structural changes of chromatin [70, 71], differential levels of DNA methylation in these RdDM mutants, including *met1*, could not affect their DDR phenotypes. In contrast, it should be noted that *DDM1* mutation affected global chromatin structures before and after gamma irradiation (Fig 6). Substantial changes in nuclear organization and chromatin structure in the *ddm1* mutants have been previously reported [72]. In eukaryotes, chromatin relaxation occurs rapidly at the DNA damage sites [12]. The relatively closed chromatin structures in the *ddm1* mutants under normal conditions could be unfavorable for the recruitment of DNA repair machinery and the subsequent HDR of DSBs after gamma irradiation [73]. Conversely, after DNA damage repair, the chromatin should rapidly return to a compaction state close to its pre-damaged level [74, 75]. The more relaxed chromatin in the *ddm1* mutants after gamma irradiation can be associated with the reduced HDR activity of DSBs as shown in SSA and possibly HR repair. Therefore, the DNA damage-sensitive phenotypes in the *ddm1* mutants would be attributed to defects in DDM1-mediated chromatin remodeling for DSB repair rather than DNA methylation itself. Some SWI2/SNF2 proteins control both nucleotide excision repair (NER)/BER and DSB repair in yeast and mammals [16], and the sensitive phenotypes of *ddm1* mutants to MMS-induced DNA damage are attributed to defects in DNA excision repair [31]. It can be assumed that DDM1 may also be involved in both DNA repair mechanisms.

In this study, we report a new function of the SWI2/SNF2 chromatin remodeler DDM1 in DSB repair. DDM1 is involved in SSA repair and probably also in HR repair by facilitating γ-H2AX and RAD51 induction. Thus, DDM1 is required for normal operation of DSB repair mechanisms in response to DNA damaging agents. The exact mechanisms by which DDM1 mediates chromatin remodeling for HDR of DSBs in the DDR should be further elucidated.

## Supporting information

S1 Fig Root phenotypes of *ddm1* mutants after gamma irradiation at five-day-old seedlings. (A) Representative bright field images of root tips. Five-day-old seedlings of Col-0 (WT) and *ddm1* mutants were irradiated with 200 Gy of gamma irradiation and were further grown for 8 days. Scale bars, 100 μm. (B) Average root width of WT and the *ddm1* mutants. Data represent average values ± SE (n = the numbers in brackets) of three independent experiments.
(TIF)

S2 Fig Phenotypes of RdDM mutants in response to gamma irradiation. Phenotypes of RdDM mutants including *ddm1-2* in response to gamma irradiation. Seedlings were grown for 14 days after gamma irradiation at different doses for 4 h. CT, control; GR, gamma radiation.
(TIF)

S1 Table Oligonucleotide primers used for qRT-PCR. (DOCX)

S1 File List of differentially expressed genes (DEGs) with more than 2-fold difference by comparison of gene expression levels between 200 Gy-treated WT and 200 Gy-treated *ddm1-2*. (XLSX)
