# Supplementary figures and images for: Mutation in DDM1 inhibits the homology directed repair of double strand breaks
Source: PLoS One. 2019 Feb 11;14(2):e0211878. doi: 10.1371/journal.pone.0211878 (PMC6370192; doi:10.1371/journal.pone.0211878)

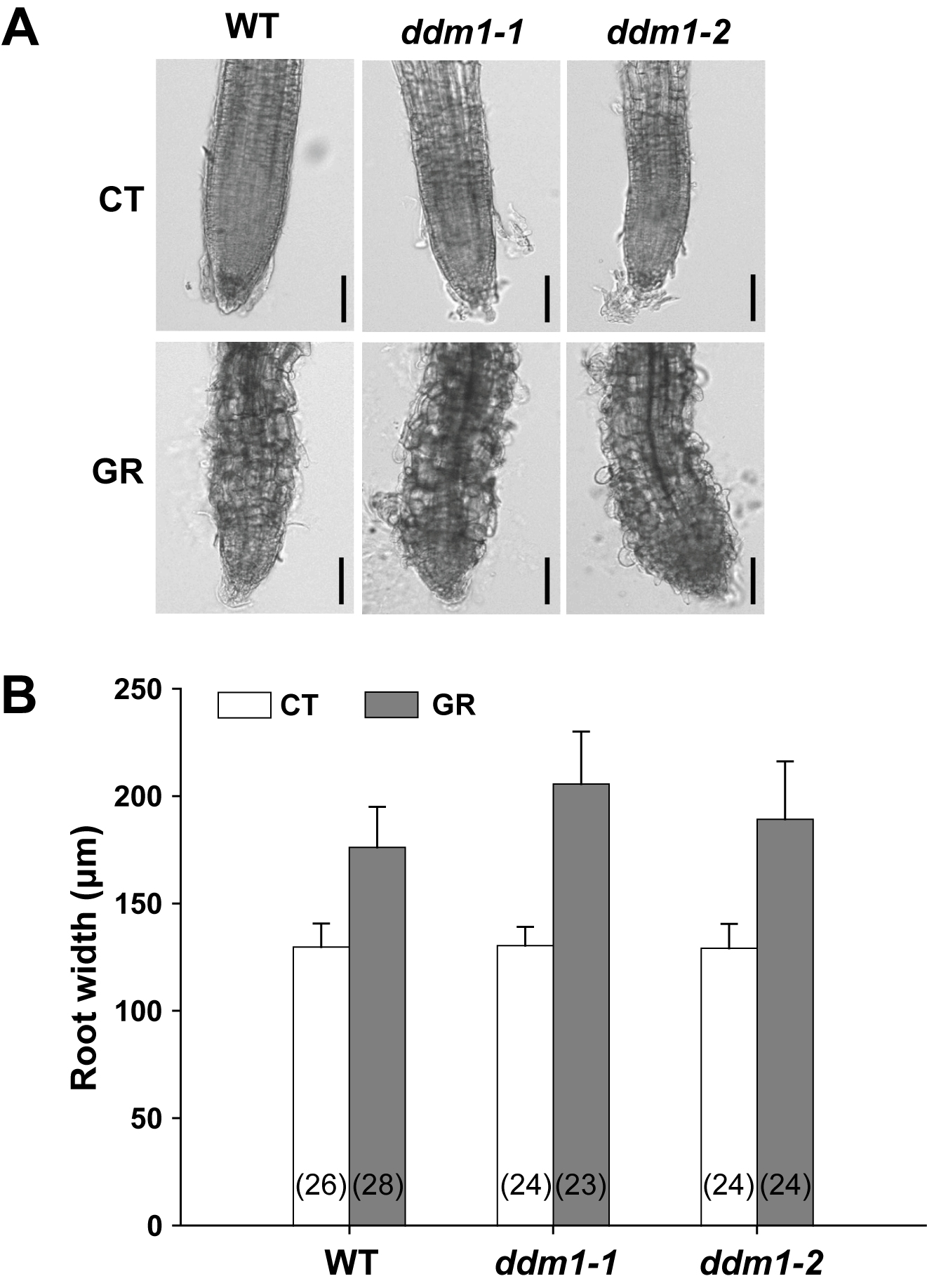

Supplement: S1 Fig — (A) Representative bright field images of root tips. Five-day-old seedlings of Col-0 (WT) and ddm1 mutants were irradiated with 200 Gy of gamma irradiation and were further grown for 8 days. Scale bars, 100 μm. (B) Average root width of WT and the ddm1 mutants. Data represent average values ± SE (n = the numbers in brackets) of three independent experiments. (TIF) [file pone.0211878.s001.tif]

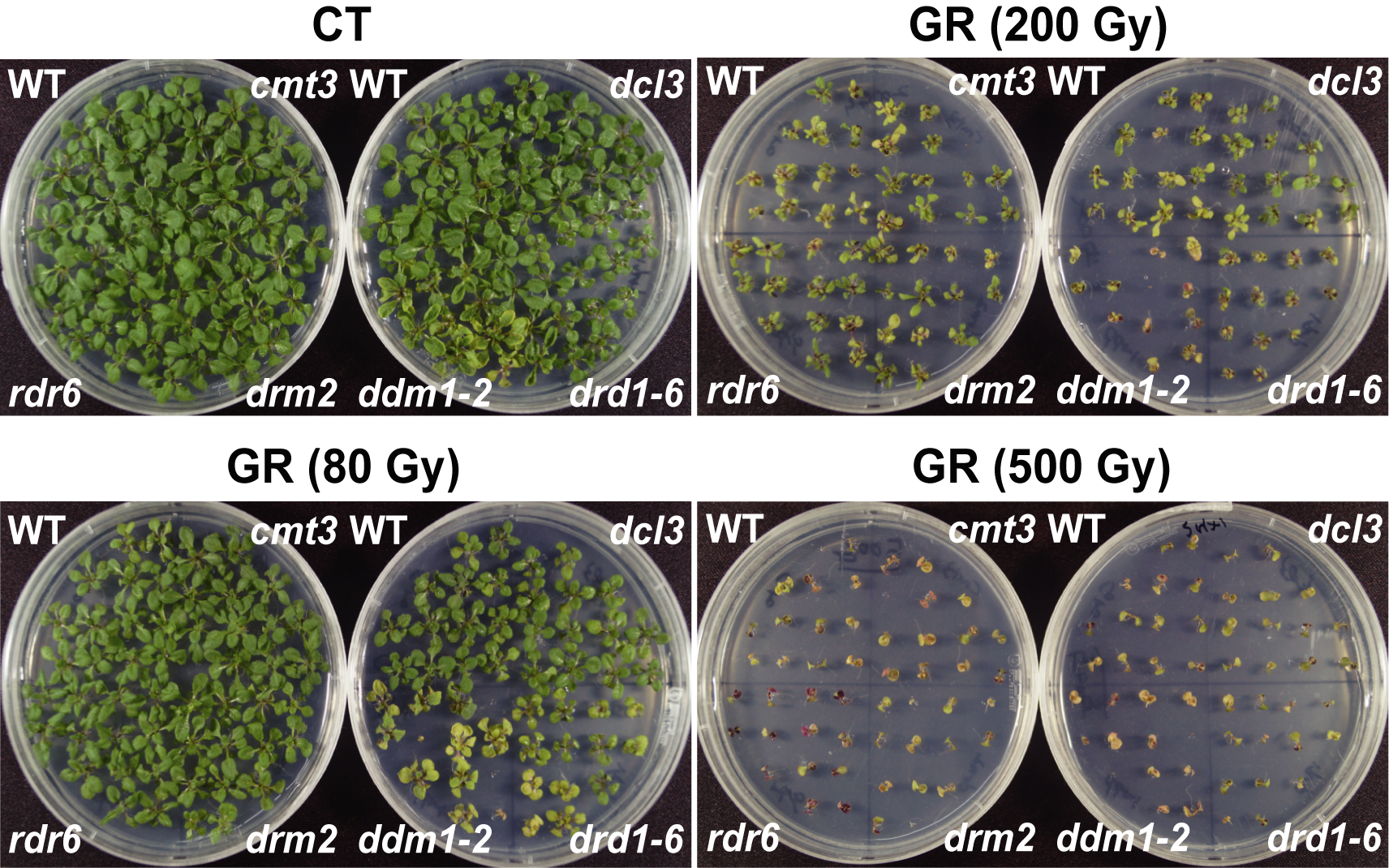

Supplement: S2 Fig — Phenotypes of RdDM mutants including ddm1-2 in response to gamma irradiation. Seedlings were grown for 14 days after gamma irradiation at different doses for 4 h. CT, control; GR, gamma radiation. (TIF) [file pone.0211878.s002.tif]
