# Supplementary material for: Mutation in DDM1 inhibits the homology directed repair of double strand breaks
Source: PLoS One. 2019 Feb 11;14(2):e0211878. doi: 10.1371/journal.pone.0211878 (PMC6370192; doi:10.1371/journal.pone.0211878)
Supplement: S1 Table — (DOCX) [file pone.0211878.s003.docx]

**S1 Table**. Oligonucleotide primers used for qRT-PCR.

| Name | Sequence (5’🡪3’) |
| --- | --- |
| *ACT2* | F: GCCCAGAAGTCTTGTTCCA  R: CTTGGTGCAAGTGCTGTGAT |
| *DML1* | F: CATTCGATGGTGTCTGATGC  R: GGGCTCTTTCATTGACTGGA |
| *REV1* | F: GCAGTGCAAGTGCTGGAATA  R: ACCAGGTAGTGTCCCGACTG |
| *XRCC4* | F: CTCGCCGAGCAATACTTAGG  R: CGTCCAAGAGAGCCGTTTAG |
| *EME1B* | F: TGTGCTGCCTCACGAATAAG  R: CTCCAATTTCCCGGATTTTT |
| *BRCA1* | F: TGAACCTGTCTCTGCGGATT  R: TGTCCCATCTGCATTCTGGT |
| *PARP1* | F: ACCCATCAGAGGCTCAAACA  R: ACGCATCTTGATTTGTTCCACA |
| *RAD51* | F: TACCGCTCTCTACAGAACAG  R: ATTCTCTCCTCTGCTCTTCC |
| *RPA1E* | F: TGGAGAAGTGACGACTGAAGC  R: ACCTCCAGTTGCGGAACAAT |
